# Androgen-Regulated Expression of Arginase 1, Arginase 2 and Interleukin-8 in Human Prostate Cancer

**DOI:** 10.1371/journal.pone.0012107

**Published:** 2010-08-11

**Authors:** Philippe O. Gannon, Jessica Godin-Ethier, Matthew Hassler, Nathalie Delvoye, Meghan Aversa, Alexis O. Poisson, Benjamin Péant, Mona Alam Fahmy, Fred Saad, Réjean Lapointe, Anne-Marie Mes-Masson

**Affiliations:** 1 Centre de recherche du Centre hospitalier de l'Université de Montréal (CRCHUM) and Institut du cancer de Montréal, Montreal, Quebec, Canada; 2 Department of Chemistry, McGill University, Montreal, Quebec, Canada; 3 Department of Surgery, CHUM, Université de Montréal, Montreal, Quebec, Canada; 4 Department of Medicine, Université de Montréal, Montreal, Quebec, Canada; Baylor College of Medicine, United States of America

## Abstract

**Background:**

Prostate cancer (PCa) is the most frequently diagnosed cancer in North American men. Androgen-deprivation therapy (ADT) accentuates the infiltration of immune cells within the prostate. However, the immunosuppressive pathways regulated by androgens in PCa are not well characterized. Arginase 2 (ARG2) expression by PCa cells leads to a reduced activation of tumor-specific T cells. Our hypothesis was that androgens could regulate the expression of ARG2 by PCa cells.

**Methodology/Principal Findings:**

In this report, we demonstrate that both ARG1 and ARG2 are expressed by hormone-sensitive (HS) and hormone-refractory (HR) PCa cell lines, with the LNCaP cells having the highest arginase activity. In prostate tissue samples, ARG2 was more expressed in normal and non-malignant prostatic tissues compared to tumor tissues. Following androgen stimulation of LNCaP cells with 10 nM R1881, both ARG1 and ARG2 were overexpressed. The regulation of arginase expression following androgen stimulation was dependent on the androgen receptor (AR), as a siRNA treatment targeting the AR inhibited both ARG1 and ARG2 overexpression. This observation was correlated *in vivo* in patients by immunohistochemistry. Patients treated by ADT prior to surgery had lower ARG2 expression in both non-malignant and malignant tissues. Furthermore, ARG1 and ARG2 were enzymatically active and their decreased expression by siRNA resulted in reduced overall arginase activity and l-arginine metabolism. The decreased ARG1 and ARG2 expression also translated with diminished LNCaP cells cell growth and increased PBMC activation following exposure to LNCaP cells conditioned media. Finally, we found that interleukin-8 (IL-8) was also upregulated following androgen stimulation and that it directly increased the expression of ARG1 and ARG2 in the absence of androgens.

**Conclusion/Significance:**

Our data provides the first detailed *in vitro* and *in vivo* account of an androgen-regulated immunosuppressive pathway in human PCa through the expression of ARG1, ARG2 and IL-8.

## Introduction

Prostate cancer (PCa) is the most frequently diagnosed cancer and third leading cause of cancer related deaths for North American men [1]. The prostate's organogenesis and carcinogenesis rely on the presence of androgens [2]. As such, the most common treatment modality for men with an advanced stage or recurrent PCa is androgen-deprivation therapy (ADT). ADT leads to the apoptosis of hormone sensitive prostate epithelial cells [3]. Unfortunately, within one to five years following ADT initiation, most patients develop hormone refractory PCa (HRPC), whose treatment remains palliative [4]. New treatment modalities, such as immunotherapy, attempt to tackle these later stages of PCa. However, current immunotherapies against PCa have resulted in limited success in the clinical settings. A detailed understanding of the tumor immunological microenvironment in prostate cancer patients should provide new insights on how to improve current immune-based protocols.

Recent data demonstrate that various immunosuppressive mechanisms are present within the prostate and may hamper the anti-tumoral immune response in the context of an immunotherapy (reviewed in [5]). Arginase 2 (ARG2) is expressed in human PCa [6] and its inhibition, concomitant with iNOS, increases the activation of tumor-infiltrating lymphocytes (TILs) [7]. While the immunosuppressive properties of arginases through the metabolism of l-arginine are well documented (reviewed in [8]), the regulation of human arginase expression, however, is currently undefined.

Androgens are known to have immunosuppressive properties, which is illustrated by the intra-prostatic inflammation following ADT [9], [10]. Gene expression analyses and murine studies suggest that androgens regulate the expression of ARG2 and other enzymes of the polyamine pathway [11], [12], [13]. Thus, considering the fundamental roles of androgens in prostate carcinogenesis and in the sculpting of the prostate's microenvironment, we evaluated whether androgens could regulate the expression of arginases by PCa cells *in vitro* and *in vivo*.

In this study, we report that PCa cell lines express both functionally active ARG1 and ARG2. Interestingly, hormone sensitive (HS) and hormone refractory (HR) tissues expressed less ARG2 than non-malignant tissues. In the HS LNCaP cell line, androgen stimulation led to the increased expression of both ARG1 and ARG2 in an androgen receptor (AR) dependant manner. This androgen-regulated expression was also observed in the primary tumor of ADT-treated patients who expressed less ARG2 in both the non-malignant tissues adjacent to the tumor and the tumor tissues compared to control patients. Finally, we discovered that IL-8 was also regulated by androgens under the control of the AR, and participated in the regulation of ARG1 and ARG2 expression. Altogether, our data provides the first detailed *in vitro* and *in vivo* account of an androgen-regulated immunosuppressive pathway in human PCa.

## Results

### ARG1 and ARG2 expression in PCa

**Figure 1:**
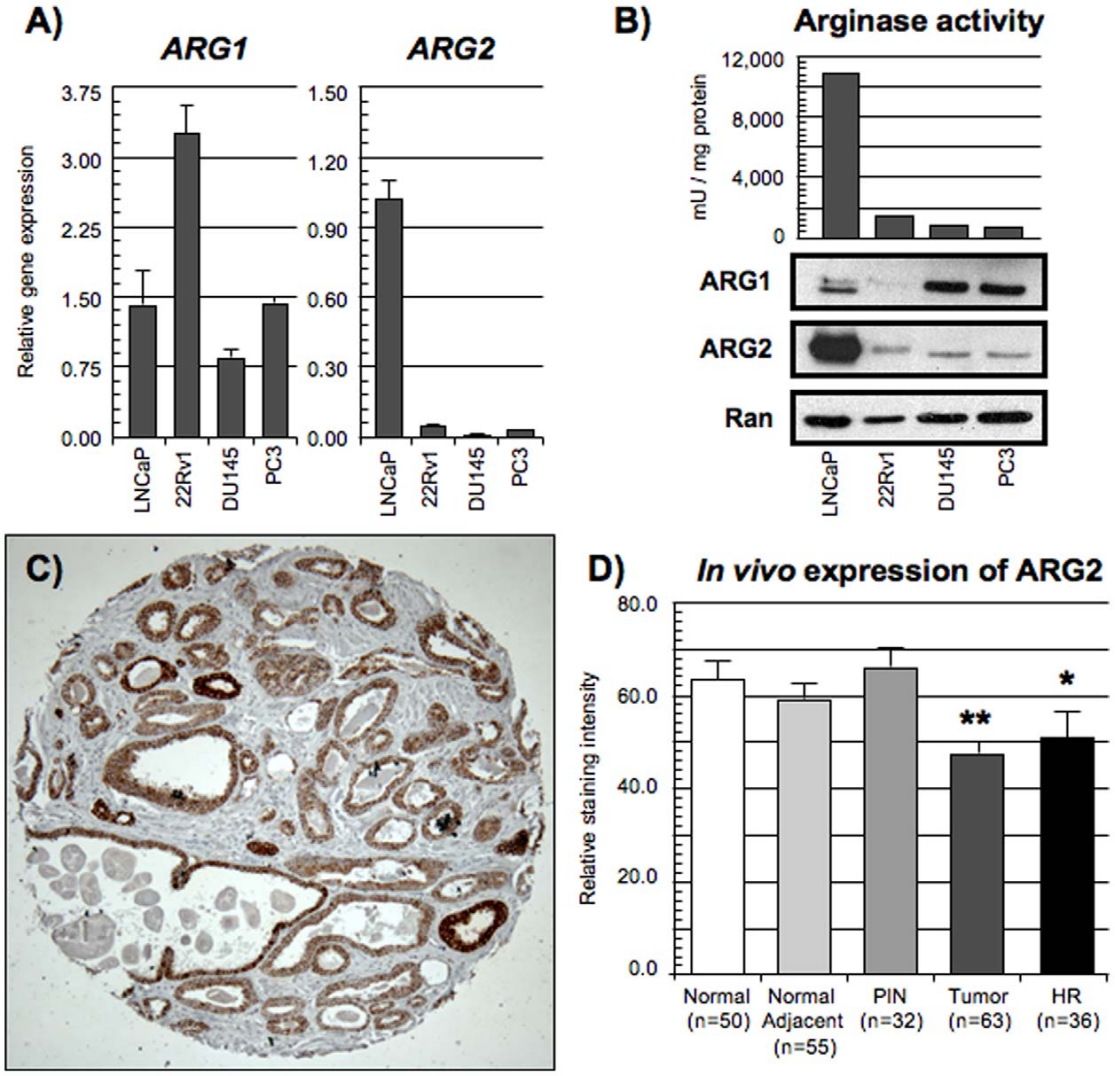
*In vitro* and *in vivo* expression of ARG1 and ARG2 in PCa. PCa cell lines (LNCaP, 22Rv1, DU145 and PC3) were maintained in RPMI supplemented with 10% FBS. A) Gene expression of *ARG1* (left panel) and *ARG2* (right panel). Mean relative expression (n = 3) with standard error of the mean (error bars). B) Top panel: Arginase activity of PCa cell lines quantified in mU/mg of proteins. Bottom panel: Western blot of ARG1 and ARG2. Ran served as loading control. C) Representative image of immunohistochemistry staining of ARG2 expression in prostatic tissue. Note that the expression of ARG2 was confined to the epithelial cells with no stromal staining. D) Quantification of ARG2 expression by immunohistochemistry in prostate specimens. *Statistically significant difference in ARG2 expression between PIN and HR tissues (p = 0.033, Mann-U). **Statistically significant difference in ARG2 expression between tumor tissues and normal tissues (p<0.001, Mann-U), non-malignant tissues adjacent to tumor (p<0.01, Mann-U) and PIN tissues (p<0.001, Mann-U).

We first evaluated arginases expression from PCa cell lines and clinical samples. Our data demonstrate that PCa cell lines express both ARG1 and ARG2. Gene expression analyses by qPCR demonstrated that *ARG1* mRNA was more abundant in the 22Rv1 cell line (Figure 1A), while ARG1 protein was slightly more expressed by the two HR PCa cell lines (Du145 and PC3) than in the LNCaP cells (Figure 1B, bottom panel). ARG1 protein expression did not correlate with the gene expression analysis suggesting a possible post-transcriptional regulation. As for ARG2 expression, the LNCaP cell line expressed the highest levels of *ARG2* mRNA (Figure 1A). Low *ARG2* mRNA expression was detected in the two HR cell lines DU145 and PC3 compared to the two HS PCa cell lines. ARG2 protein abundance correlated with the qPCR results with LNCaP cells expressing significantly more ARG2 than the other three cell lines (Figure 1B, bottom panel). Furthermore, LNCaP cells had the highest arginase activity suggesting that ARG2 is the predominant enzyme with regards to arginase activity of PCa cells (Figure 1B, top panel).

Expression of the ARG2 protein was evaluated in clinical samples by immunohistochemistry on three different tissue micro-arrays (TMAs) regrouping prostate samples from a cohort of 99 PCa patients and 50 normal prostate obtained from autopsies. We did not evaluate ARG1 protein expression as, in our hands, anti-ARG1 antibodies tested were not suitable for immunohistochemistry on archived formalin-fixed paraffin-embedded tissues. We observed that ARG2 expression was restricted to the prostate epithelium and absent from the stroma (Figure 1C). ARG2 was statistically significantly less expressed in tumor tissues compared to normal (p<0.001, Mann-U), to non-malignant normal adjacent (p<0.01, Mann-U) and to prostate intraepithelial neoplasia (PIN) tissues (p<0.001, Mann-U) (Figure 1D). HR tissues also expressed less ARG2, although only significantly different from PIN tissues (p = 0.033, Mann-U). There was no correlation between ARG2 expression within the normal adjacent and tumor tissues (Table S1). Finally, we evaluated if the ARG2 expression correlated with clinico-pathological parameters such as Gleason Score, pre-operative prostate specific antigen (PSA) levels and biochemical recurrence. Our results show that ARG2 expression within the normal adjacent tissue inversely correlated with vesicle seminal invasion (Table S1). Altogether, these *in vitro* and *in vivo* data demonstrate the differential expression of ARG1 and ARG2 between various stages of PCa progression, independently of the HR status.

### Androgen-regulated expression of ARG1 and ARG2

**Figure 2:**
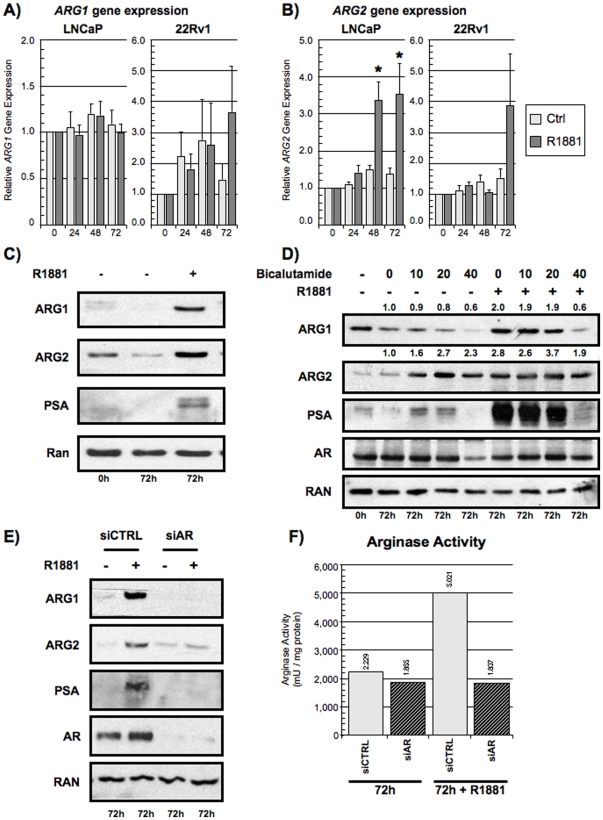
Androgen-regulated expression of ARG1 and ARG2. A-B) LNCaP cells (left panels) and 22RV1 (right panels) were stimulated over a period of 72 hours with 10 nM R1881 following a 72 hour incubation period in charcoal-stripped media and the gene expression of A) *ARG1* and B) *ARG2* analyzed by qPCR. Control (light gray bars) and R1881-stimulated (black bars). *Statistically significant difference (p<0.05, Mann-U). Mean relative expression (n = 4) with standard error (error bars). C) Increased protein expression of both ARG1 and ARG2 following R1881 stimulation by Western blot. LNCaP cells were stimulated with 10 nM R1881 as previously described. PSA served as positive control. Representative experiment, (n = 6). D) Inhibition of AR activity with bicalutamide (Casodex). LNCaP cells were stimulated with R1881 for 72 hrs as previously described in the presence of increasing doses of bicalutamide (0, 10, 20 and 40 µM). ARG1 and ARG2 expression levels were evaluated by Western blot. Representative experiment is shown, (n = 3). Note the agonist effect of bicalutamide in the absence of R1881 illustrated by an increased PSA and ARG2 expression. E) Inhibition of AR expression by siRNA. LNCaP cells were transfected as previously described. AR, ARG1 and ARG2 expression levels were evaluated by Western blot. Representative experiment is shown, (n = 4). F) Arginase activity was quantified in mU/mg of proteins. LNCaP cells were transfected with siCTRL (light gray bars) or siAR (black bars) and then stimulated with R1881 for 72 hrs as previously described. Representative experiment, (n = 3).

The differential expression of ARG1 and ARG2 between the HS and HR PCa cell lines led us to investigate the regulatory roles of androgens in arginase expression. To do so, we evaluated arginases gene and protein expression following androgen stimulation. *ARG1* mRNA expression was not statistically significantly upregulated in either LNCaP or 22RV1 cell lines following R1881 stimulation (Figure 2A). However, in LNCaP cells, *ARG2* mRNA expression was increased at 48 hours (p = 0.002, Mann-U) and at 72 hours (p = 0.016, Mann-U) following the R1881 stimulation (Left panel, Figure 2B). The overexpression of *ARG2* in 22RV1 was not statistically significant (p = 0.248, Mann-U) (Right panel, Figure 2B). In fact, *ARG2* expression correlated with the higher androgen sensitivity of LNCaP cells compared to 22RV1 (Figure S1A). As such, LNCaP cells were used for further experiments. Corroborating the PCR data, Western blots from LNCaP cells demonstrated that the R1881 stimulation increased ARG2 protein expression (Figure 2C). Interestingly, although no significant changes were observed in *ARG1* mRNA expression in LNCaP cells treated with R1881, ARG1 protein expression was significantly increased. We did not observe any increases in ARG1 or ARG2 protein expression in DU145 and PC3 stimulated with 10 nM of R1881 (Figure S1B).

### The AR is implicated in ARG1 and ARG2 expression

As our results suggest that androgens regulate arginase expression, we evaluated the contribution of the AR. We inhibited AR activity with the non-steroidal anti-androgen bicalutamide (Casodex) (Figure 2D). We noted a decreased expression of ARG1 with the highest concentration (40 µM) of bicalutamide following R1881 stimulation. The androgen induction of ARG2 was not blocked, even at the highest concentration of bicalutamide. As previously documented [14], we observed that bicalutamide had AR-agonist activity in LNCaP cells cultured in the absence of androgens. There was an R1881-independant induction of PSA and ARG2 expression in LNCaP cells stimulated with 20 µM and 40 µM of bicalutamide in the absence of androgens. In this same condition, bicalutamide caused a decrease in ARG1 expression. These results suggest that ARG1 expression may be more sensitive to AR inhibition than ARG2, whose expression was induced by the agnostic effect of the AR inhibitor.

We decided to further inhibit the AR by blocking the AR expression in LNCaP cells using siRNA. The presence of siRNA against the AR resulted in a significant inhibition of AR expression and in a reduced PSA expression following R1881 stimulation (Figure 2E). Both the ARG1 and ARG2 induction following R1881 stimulation were inhibited by the siRNA treatment, which translated in the absence of an upregulation in arginase activity (Figure 2F). These results suggest that the AR regulates the expression of ARG1 and ARG2 *in vitro* and that ARG1 and ARG2 have a different sensitivity to AR inhibition.

### Diminished ARG2 expression in PCa patients following ADT

**Figure 3:**
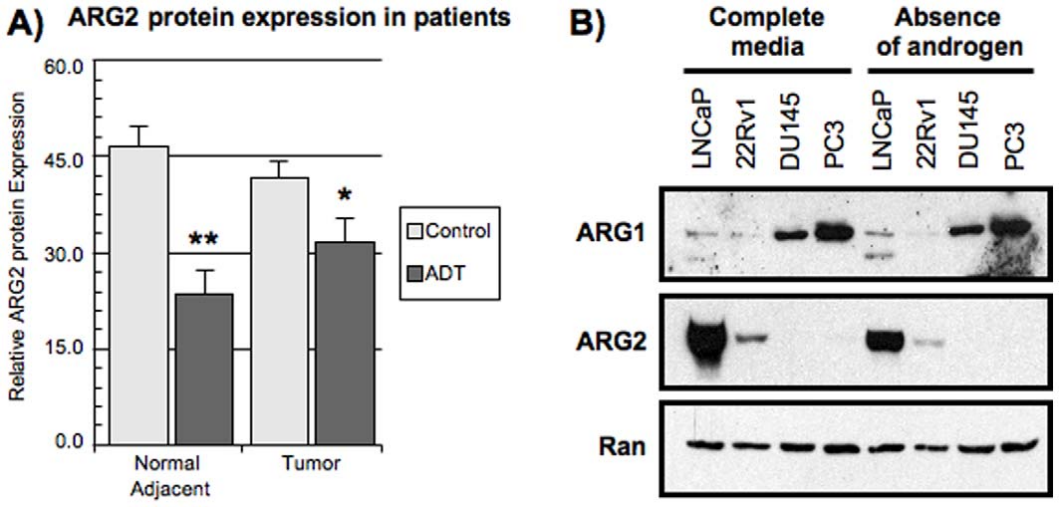
Reduced ARG2 expression following ADT. A) Analysis of androgen-regulated ARG2 expression in PCa patients by immunohistochemistry. Control patients (light gray bars, n = 40) and ADT-treated patients (black bars, n = 35). B) Decreased ARG2 protein expression in the absence of androgens *in vitro* determined by Western blot. Ran served as loading control. PCa cell lines (LNCaP, 22Rv1, DU145 and PC3) were maintained in RPMI 10% FBS or in RPMI supplemented with 10% charcoal stripped FBS for 7 days (n = 3). Note that ARG1 expression did not vary but that ARG2 was reduced in LNCaP and 22Rv1 cells in the absence of androgen.

Based on our *in vitro* data, we hypothesized that androgens might modulate ARG2 protein expression in PCa patients as well. We observed that, compared to control cancer patients (surgery only), ADT-treated patients (ADT prior to surgery) had significantly lower ARG2 expression in both the non-malignant tissues adjacent to the tumor (46.4 vs 23.5 relative units; p<0.001, Mann-U) and the tumor tissues (41.7 vs 31.5 relative units; p<0.01, Mann-U) (Figure 3A). We also observed that androgen deprivation *in vitro* could decrease ARG2, but not ARG1 protein expression, in LNCaP and 22RV1 cells cultured for seven days in the absence of androgens (Figure 3B). Taken together, these results suggest that androgens regulate the expression of ARG2 *in vivo* in PCa patients as ADT reduces ARG2 expression.

### ARG1 and ARG2 are metabolically active

**Figure 4:**
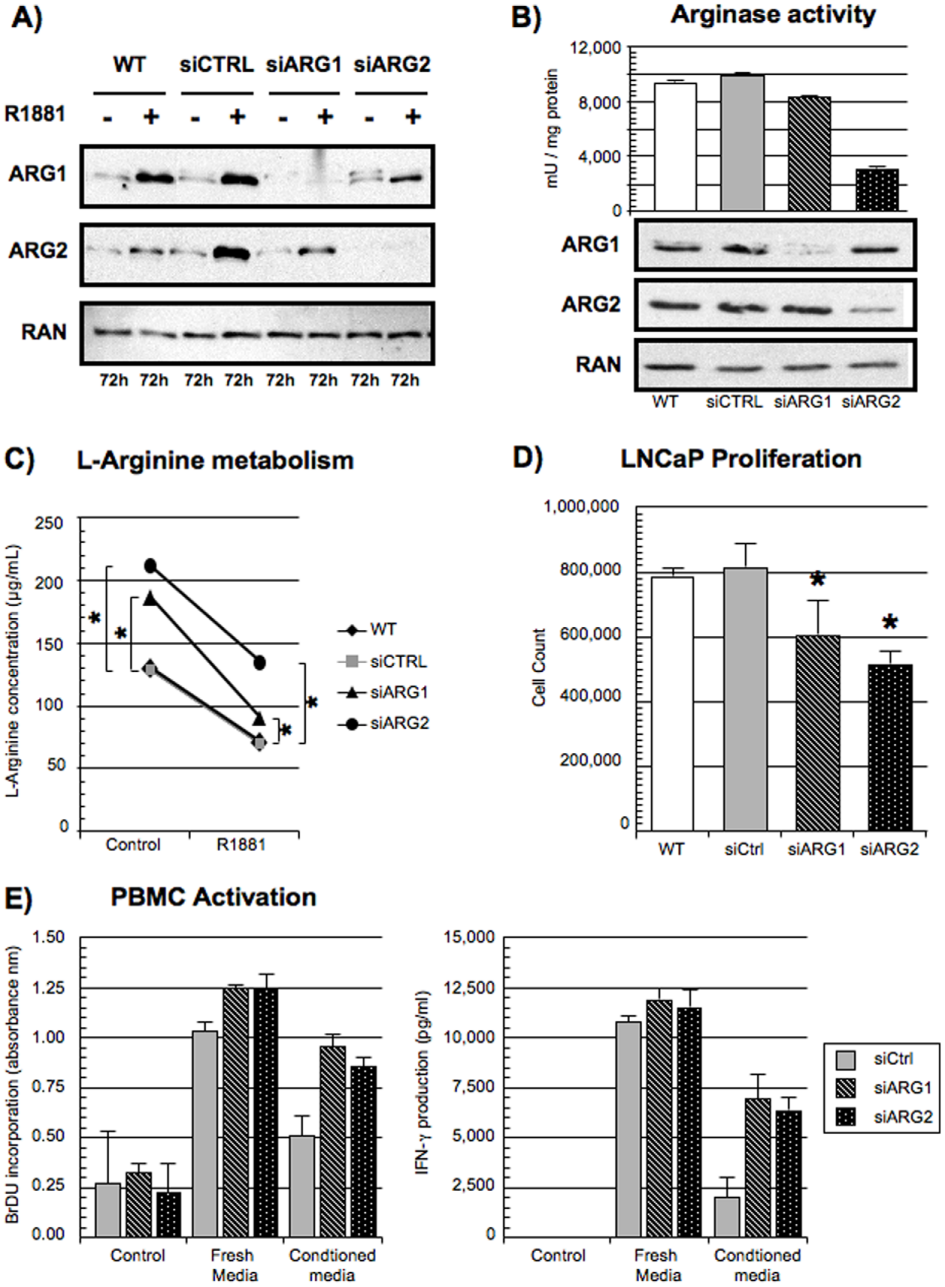
ARG1 and ARG2 are metabolically active. LNCaP cells were transfected with either a siCTRL or a cocktail of three siRNA against the ARG1 or ARG2. Post-transfection (24 hours), cells were plated in charcoal-stripped serum supplemented media for 72 hours and then stimulated for 72 hours with 10 nM R1881. A) siRNA inhibition of ARG1 and ARG2 expression was evaluated by Western Blot. Representative experiment is shown, (n = 4). B) Decreased arginase activity following transfection with siARG1 or siARG2 in LNCaP cells. The corresponding Western blot is shown in the bottom panel. Representative experiment is shown, (n = 3). C) Decreased metabolism of l-arginine in the absence of arginase expression. Conditioned media of LNCaP cells transfected with siCTRL, siARG1 or siARG2 were analyzed by HPLC for l-arginine concentration. The conditioned media analyzed by HPLC were from the LNCaP cells presented in Figure 4A. *Statistically significant difference (p<0.05, Mann-U). D) Decreased proliferation of LNCaP cells in the absence of arginase expression. LNCaP cells were transfected as previously described. Proliferation was measured by cell count 96 hours post-transfection. *Statistically significant difference (p<0.05, Mann-U), (n = 3). E) Inhibition of ARG2 expression causes increased PBMC proliferation and activation. PBMCs from normal donors were activated with anti-CD3 (OKT3, 1 µg/ml) with or without IL-2 in the presence of fresh media or conditioned media of LNCaP cells transfected with either control, siCTRL, siARG1 or siARG2 as previously described. Control PBMCs were incubated with IgG isotype control (1 µg/ml) and with PBS instead of IL-2. Left panel: PBMC proliferation was quantified by BrdU incorporation following 120 hours of OKT3 and IL-2 stimulation. Mean absorbance (n = 4) is shown with standard error (error bars). Right panel: PBMC secretion of IFN-γ quantified by ELISA. Same experiment as previously described, but the PBMCs were activated for 24 hours without IL-2. Representative expression is shown (n = 4) with standard error of the mean (error bars).

To evaluate whether ARG1 and ARG2 expressed by LNCaP cells were metabolically active, we inhibited the expression of either ARG1 or ARG2 by siRNA. Compared to a siCTRL, both siRNA significantly inhibited ARG1 or ARG2 expression (Figure 4A). Inhibition of either ARG1 or ARG2 resulted in diminished arginase enzymatic activity (Figure 4B). By HPLC, we then determined the impact of the inhibition of ARG1 and ARG2 expression on the metabolism of l-arginine by LNCaP cells. The absence of either ARG1 or ARG2 led to higher concentrations of l-arginine in the conditioned media suggesting a lower metabolism of l-arginine by LNCaP cells (Figure 4C). Moreover, we noted that R1881 stimulation led to a decreased concentration of extracellular l-arginine, which corroborates our results demonstrating an increased arginase expression following androgen stimulation. We evaluated the expression of nitric oxide synthase (NOS), also known to metabolize l-arginine. However, we did not observe the expression of iNOS or nNOS (protein) as well as no production of NO (function) in our model (data not shown).

As arginases are implicated in the polyamine synthesis pathway necessary for cellular proliferation, we evaluated the impact of ARG1 and ARG2 on cell growth. We observed that inhibition of either ARG1 or ARG2 expression resulted in a lower proliferation of LNCaP cells maintained in complete media (p = 0.02 and p = 0.01, respectively for siARG1 and siARG2) (Figure 4D). Furthermore, in order to study whether ARG1 and ARG2 expression by LNCaP cells affected their immunosuppressive potential, PBMCs from healthy donors were activated in the presence of conditioned media from LNCaP+siCTRL or LNCaP+siARG1 or LNCaP+siARG2. The inhibition of either ARG1 or ARG2 translated into increased PBMC proliferation as quantified by BrdU incorporation (Figure 4E, left panel). This increased proliferation was associated with an elevated IFN-γ secretion by PBMCs as measured by ELISA (Figure 4E, right panel). No significant variations in the secretion of IL-2 or IL-10 were observed (data not shown). Finally, we evaluated whether the ARG2 expression correlated with the immune cell infiltrate of the primary tumor that we recently published [10]. We noted that ARG2 expression did inversely correlate with the infiltration of T lymphocytes and macrophages within the prostate (Table S2). Collectively, these results suggest that ARG1 and ARG2 expressed by LNCaP cells are enzymatically active and participate in important physiological processes such as cellular proliferation and tumor-derived immunosuppression.

### IL-8 induction of ARG1 and ARG2 expression

**Figure 5:**
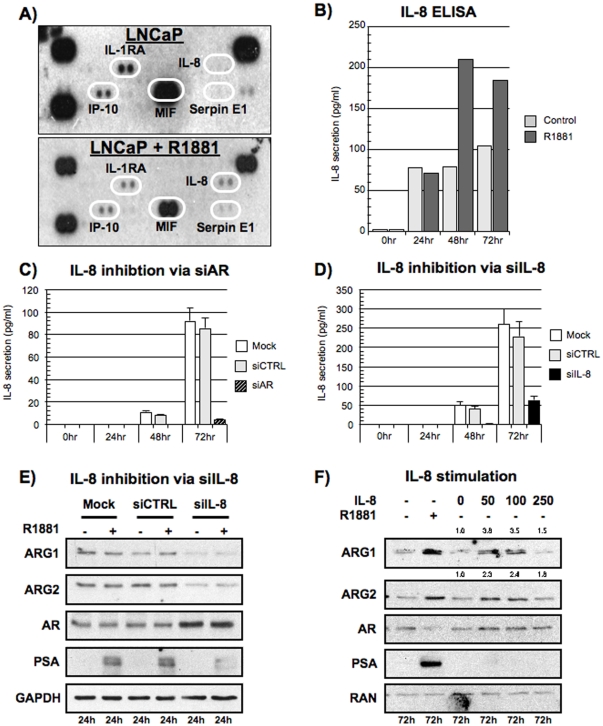
Androgens induced Interleukin-8, which in turn promotes ARG1 and ARG2 expression. Evaluation of the cytokine expression profile of LNCaP cells following R1881 stimulation. A) Conditioned media of LNCaP cells stimulated as previously described were analyzed with a Proteome Profiler™ (R&D Systems). B) Conditioned media of LNCaP cells stimulated over time with either ethanol control (light gray bars) and R1881 (black bars) were analyzed for the production of IL-8 by ELISA. The representative experiment showed was performed with the same conditioned media used for the Proteome Profiler analysis in 5a, (n = 3). C) Quantification of IL-8 secretion by LNCaP cells transfected with siAR and stimulated with R1881 as previously described. Representative experiment is shown, (n = 3). D) Quantification of IL-8 secretion by LNCaP cells transfected with siIL-8 and stimulated with R1881 as previously described. Representative experiment is shown, (n = 3). For 5b and 5c, there was no IL-8 secretion detected in the absence of R1881 stimulation. E) Expression of ARG1 and ARG2 in LNCaP cells following transfection of siIL-8 and R1881 stimulation for 24 hours. Representative experiment is shown, (n = 3). F) LNCaP cells were plated in charcoal-stripped serum supplemented media for 72 hours and for 24 hours in serum-free RPMI. Cells were then stimulated for 72 hours with 10 nM R1881 or with 50, 100 or 250 ng/ml of IL-8 in serum-free RPMI. ARG1 and ARG2 expression levels were detected by Western blot. Representative experiment, (n = 3). Note the induction of both ARG1 and ARG2 at 50 and 100 ng/ml of IL-8 concentration in the absence of R1881.

As arginases are well-documented to participate in the sculpting of the tumor immunological microenvironment [15] and that cytokines are known to induce arginase expression in murine models [16], we assessed whether cytokines could also regulate arginase expression in human PCa. The cytokine expression profile of LNCaP cells stimulated with 10 nM of R1881 was qualitatively evaluated using a Proteome Profiler cytokine array (R&D Systems) (Figure 5A). The proteomic data illustrated that the R1881-stimulated LNCaP cells had increased expression of IL-8 and Serpin E1 (Figure S2A). We further investigated the role of IL-8 in arginase expression as IL-8 has been recently linked to the expression of androgen-regulated genes in PCa [17]. We thus quantified the elevated expression of IL-8 in LNCaP cells following R1881 expression (Figure 5B). This IL-8 induction was dependent on the AR as the inhibition of AR expression by siRNA prevented IL-8 secretion following androgen stimulation (Figure 5C). We then evaluated whether inhibition of IL-8 could diminish ARG1 and ARG2 expression following R1881 stimulation. Using a siRNA against IL-8, we could significantly diminish IL-8 secretion (Figure 5D). This reduced IL-8 production was associated with a reduction of ARG1 and ARG2 24 hrs following R1881 stimulation (Figure 5E). The treatment of LNCaP cells with siIL-8 also translated to a decrease in arginase activity (Figure S2B). Finally, we stimulated androgen-deprived LNCaP cells with increasing concentration of exogenous IL-8 for 72 hrs and monitored the expression of ARG1 and ARG2. By Western blot analysis, we observed that both 50 ng/ml and 100 ng/ml of IL-8 induced the expression of ARG1 and ARG2 when compared to control LNCaP cells (Figure 5F). The decrease in ARG1 and ARG2 protein expression with 250 ng/ml of IL-8 correlated with IL-8 induced cellular toxicity. We also observed an induction of *ARG2*, but not *ARG1*, gene expression after a 24 hr stimulation (Figure S2C). Finally, as it was previously reported that phenol red could activate the AR [18], we also stimulated LNCaP cells with IL-8 in phenol red-free RPMI media. These control experiments demonstrated that IL-8-dependent induction of ARG1 and ARG2 could occur in the absence of phenol red (Figure S2D). Taken together, the data clearly shows that androgens regulate the expression of IL-8, which on its own can induce the expression of both ARG1 and ARG2.

## Discussion

A more thorough understanding of the prostate immunological microenvironment mechanisms may improve the clinical efficacy of current immunotherapies against PCa. We and others have shown that ADT leads to drastic changes in the prostate immunological microenvironment [9], [10]. The arginase pathway participates in the development of an immunosuppressive state within the primary tumor of PCa patients [7]. However, the regulation of arginase expression by PCa cells remains undefined.

In this report, we observed that androgens induced the expression of both ARG1 and ARG2 in HS PCa cell lines. The AR was implicated in this regulation as both bicalutamide and siAR transfection prevented ARG1 and ARG2 overexpression following R1881 stimulation. Reciprocally, androgen deprivation and ADT reduced ARG2 expression *in vitro* and in the primary tumor of PCa patients, respectively. LNCaP cells expressed enzymatically functional ARG1 and ARG2 which, once their protein expression was inhibited, caused a decrease in cellular proliferation and in their immunosuppressive potential. Finally, we showed that IL-8 was also regulated by R1881 and could stimulate the expression of ARG1 and ARG2 independently of androgen. Altogether, our results provide the first mechanistic evidence of an androgen-driven immunosuppressive pathway in PCa through the expression of ARG1, ARG2 and IL-8 by PCa cells.

We demonstrate that PCa cells express both ARG1 and ARG2. ARG2 was predominantly expressed by HS PCa cell lines and by non-malignant prostate tissues. These results corroborate published data describing a lower ARG2 expression in androgen-insensitive PCa cell lines (DU145 and PC3) and in the tumor and HR tissues of PCa patients [6], [19]. However, to our knowledge, we are the first group to study the expression of ARG1 by PCa cells and define mechanistic consequences leading to its androgen-regulated induction. Similar to ARG2, inhibition of ARG1 expression led to decreased tumor cell proliferation, reduced l-arginine metabolism and reduction of their immunosuppressive potential. Based on the protein expression (Figure 1B, bottom panel) and on the arginase activity of PCa cells (Figure 1B, top panel), our data suggest that ARG2 may nonetheless have a more prominent role than ARG1 in the arginase activity potential of PCa cells [20].

Furthermore, our data showed that ARG1 and ARG2 were differentially regulated by androgens. Contrary to ARG2 gene and protein expression, we clearly demonstrated that the gene and protein expression of ARG1 did not correlate. This suggests that androgens may influence a post-transcriptional regulation of ARG1 as it was previously reported in xenopus [21] and in yeast models [22]. Since ARG2 expression is localized to mitochondria, we evaluated whether cellular proliferation independent of androgens could induce ARG2 expression in LNCaP cells. In a proliferation assay with EGF instead of R1881, no ARG2 induction was observed (data not shown). Collectively, our results reveal that, although both induced by R1881, the signaling pathways leading to ARG1 and ARG2 expression differ for the two enzymes and need to be further examined.

The implication of an androgen-regulated expression of ARG1 and ARG2 in prostate carcinogenesis requires further investigation. Arginase expression and polyamine synthesis are elevated in PCa [23], [24] and associated with tumor grade [25]. A high arginase activity correlates with increased proliferation of breast cancer [26], colon cancer [27] and kidney cell lines [28]. However, we observed that tumor or HR tissues express less ARG2 than non-malignant tissues. It is possible that tumor cells do not acquire the expression of these enzymes as a mean to further exploit their immunosuppressive potential, an aspect associated with tumor progression. In fact, since the prostate is the organ with the highest polyamine production, arginase expression by prostate cells may precede the development of cancer, as polyamine production is essential for the proliferation of prostate cells. Thus, the immunosuppressive advantage gained by prostate cells may be secondary to the proliferative role played by the arginases. From our data and that of others, we hypothesize that arginase may be implicated in the earlier hormone-sensitive stages of prostate carcinogenesis by promoting cancer cell proliferation and the development of an androgen-regulated immunosuppressive environment.

Finally, we observed that IL-8 was upregulated following androgen stimulation and could induce the expression of ARG1 and ARG2. IL-8 mediates its effects through the activation of two high-affinity G-protein coupled receptors, CXCR1 and CXCR2 [29], both of which are expressed by LNCaP cells [30], [31]. It is important to note that expression of ARG1 and ARG2 following IL-8 stimulation was not as substantial as with R1881 stimulation suggesting that other androgen-regulated pathways could be involved. Altogether, this is the first indication that the expression of IL-8 is regulated by androgens and that arginases can be regulated by cytokine in human cancer cells.

### Conclusion

Our data demonstrate that androgens regulate the expression of both ARG1 and ARG2 in HS PCa cell lines and in PCa patients in an AR-dependent manner. ARG1 and ARG2 are enzymatically active and their inhibition results in reduced l-arginine metabolism, cell growth and immunosuppressive potential. We found that IL-8 secreted by LNCaP cells was also regulated by androgens and could on its own promote the expression of ARG1 and ARG2. Collectively, the results presented in this report suggest that androgens actively participate in the development of an immunosuppressive microenvironment within the prostate through the expression of ARG1 and ARG2. A better understanding of the expression of immunosuppressive pathways at specific stages of PCa progression may eventually provide new insights for improving current immunotherapeutic strategies.

## Materials and Methods

### Cell Culture

LNCaP, 22Rv1, DU145 and PC3 cell lines were obtained from ATCC (MD, USA). All cell lines were maintained as previously described by our group [32]. For R1881 stimulation, cells were plated at 600,000 cells per 60 mm petri dish and incubated for an initial 72 hours in 10% (v/v) charcoal-stripped fetal calf serum (FCS)-supplemented RPMI 1640, which eliminates all steroid hormones from the serum. Afterwards, the cells were washed with PBS and cultured in fresh 10% charcoal-stripped FCS-supplemented RPMI 1640, with either 10 nM R1881 or ethanol (control) [32]. As control experiments, phenol red-free RPMI was purchased from Wisent (St-Bruno, QC) and was supplemented with 20 µM of L-glutamine (Wisent). Conditioned media, protein and RNA were extracted at 0, 24, 48 or 72 hours following the R1881 stimulation. For IL-8 stimulation, LNCaP cells were plated in charcoal-stripped serum supplemented media for 72 hours followed by 24 hours in serum-free RPMI. Cells were then stimulated for 72 hours with either 10 nM R1881 or with 100 ng/ml IL-8 (PeproTech, Rocky Hill, NJ) in serum-free RPMI. siRNA targeting the AR, ARG1, ARG2 and IL-8 as well as the RISC-free siGLO fluorescent siRNA control were purchased from Dharmacon (Chicago, IL). When LNCaP cells reached 80% confluence in a 100 mm petri dish they were transfected as recommended by the manufacturer using the Dharmafect 2 transfection reagent. Cells were incubated for 24 hrs after which they were seeded as described for the R1881 stimulation.

### siRNA sequences

The siRNAs were all purchased from Dharmacon and consisted of pools of four sequences.

AR siRNA sequences (catalog # M-0034-00-00): GGAACUCGAUCGUAUCAUUUU; CAAGGGAGGUUACACCAAAUU; UCAAGGAACUCGAUCGUAUUU; GAAAUGAUUGCACUAUUGAUU. ARG1 siRNA sequences (catalog # L-009922-00): GGACUGGACCCAUCUUUCA; GGGCGGAGACCACAGUUUG; GGGCUACUCUCAGGAUUAG; GAAGUAACUCGAACAGUGA. ARG2 siRNA sequences (catalog # M-091965-00): GAUCAAACCUUGUAUCUCUUU; UCAGAGAACUACAGGAUAAUU; GAACUAUGAUAUCCAGUAUUU; GGACUAACCUAUCGAGAAGUU. IL-8 siRNA sequences (catalog # L-004756-00): GCAUAAAGACAUACUCCAA; CCACCACACUGCGCCAACA; GCCAAGGAGUGCUAAAGAA; UGAAGAGGGCUGAGAAUUC.

### Antibodies

The following antibodies were purchased from Santa Cruz (Santa Cruz, CA): anti-ARG1 (BC9, sc-47715), anti-ARG2 (L-20, sc18357), anti-PSA (C-19, sc-7638), anti-RAN (C-20, sc-1146). The anti-AR (Ab-1) was purchased from LabVision/NeoMarkers (Fermont, CA) and the anti-GAPDH (ab9485) from AbCam (Cambridge, MA).

### Gene and Protein Expression

Quantitative real-time PCR (qPCR) analyses were performed as previously described by our group [33]. *RAN* served as the housekeeping gene as we found that its expression was not sensitive to R1881 stimulation. Relative mRNA of candidate gene/*RAN* ratios were calculated using the method described by Pfaffl *et al.* [34]. Fold change was calculated relative to the mock treated control. Western blotting of proteins extracted in non-denaturing buffer was performed as previously described by our group [35].

### Arginase Activity

Arginase activity was quantified as previously described [36]. Briefly, a solution of 10 mM MnCl_2_/50 mM Tris/HCl at pH 7.5 was added to whole cell extracts. Following an incubation at 55°C for 60 mins, 25 µl of 0.5 M arginine pH 9.7 was added to the samples and further incubated for 60 mins at 37°C. The arginine hydrolysis reaction was stopped by adding H_2_SO_4_/H_3_PO_4_/H_2_O at a ratio of (1∶3∶7, v/v/v). The samples were then boiled at 100°C for 15 mins following the addition of 9% ISPF and read at 540 nm. Using a standard curve, arginase activity was reported as mUnits/mg of protein.

### Immunohistochemistry on PCa TMAs

Four different tissue microarrays (TMAs) were used in this study. The first TMA contained 50 normal prostate specimens obtained from 39 autopsied patients without PCa. The second TMA contained non-malignant tissue adjacent to tumor (n = 55), prostate intra-epithelial neoplasic (PIN) tissue (n = 32) and HS tumor tissue (n = 63) from 63 patients who had undergone radical prostatectomy [37]. The third TMA contained HR tumor tissues obtained by trans-urethral resection of the prostate (TURP) from 36 patients collected subsequent to hormone therapy failure [38], [39]. Finally, the fourth TMA contained prostate specimens obtained from 35 patients who were treated by ADT prior to radical prostatectomy (ADT group) and 40 Gleason-matched control patients who were only treated by radical prostatectomy, as previously described [10]. For each patient, a total of four tumor cores and two normal adjacent cores were spotted on duplicate TMAs. Cell pellets of each PCa cell line (RWPE, LNCaP, 22Rv1, DU145 and PC3) were spotted on each array and served as internal staining controls. Ethics approval for this study was obtained from the local ethics review committee, the Comité d'éthique du Centre de recherche du Centre Hospitalier de l'Université de Montréal (CRCHUM). Furthermore, informed written consent was obtained from all participants involved in this study, including from the families of the autopsied patients.

Immunohistochemical staining was done as previously described by our group [39], [40], [41]. Briefly, the 90 min primary antibody incubation was followed by 30 min incubation with an anti-mouse HRP-coupled secondary antibody (sc-2005, Santa Cruz). Positive signals were developed with diaminobenzidine (DAB) (Dako Cytomation, Mississauga, On, Canada) and the nuclei were counterstained with haematoxylin. High-resolution digital images of each TMA were generated using a whole-slide scanner (SanScope XT automated high-throughput scanning system) from Aperio (Vista, CA). Two independent observers evaluated the intensity (0, 1+, 2+, 3+) and the percentage of positively stained cells. For each core a value corresponding to the intensity multiplied by the percentage of stained cells was calculated and reported for statistical analysis.

### Quantification of l-Arginine concentration by HPLC

Perchloric acid (150 µl) was added to conditioned media (150 µl), which was then vortexed and shook for 10 min. The samples were then centrifuged (13,000 rpm) for 20 min and 240 µl of supernatant were transferred into an amber eppendorff tube. This solution containing the l-arginine was thus essentially cleared of cellular proteins [42]. The supernatant was then neutralized with 60 µl of 3 M NaOH and buffered to pH 9.0 using 180 µl of borate buffer. To this solution, 10 µl of 0.1 M NaCN and Naphthalene-2,3-dicarboxaldehyde (NDA) were added and shaken for 20 min before injection into the HPLC. All samples were run on a Varian Pursuit C18 column 250×4.6 mm with the following three solvents: Solvent A: 100 mM triethylammonium acetate (TEAA) buffered to pH 7.0 with 5% acetonitrile (ACN) in milli-Q water; Solvent B: 60% ACN in Solvent A; Solvent C: 100% ACN. A series of l-arginine standards were made ranging from 0 to 2.58×10^−4^ g/ml. Each standard was done in triplicate and was functionalized with NDA to determine the retention time of l-arginine and the area under the peak corresponding to l-arginine at specific concentrations. Samples were monitored at 260 nM and 420 nM to identify which samples had been functionalized with the NDA. Peaks that appear at 420 nM correspond to substances that have a primary amine available to react.

### Lymphocyte activation

PBMCs from healthy donors were isolated from whole blood by Ficoll gradient using lymphocyte-separating medium (Wisent, St-Bruno, Qc, Canada). PBMCs (150,000) were incubated in a 96-well flat-bottomed plate with 1 µg/ml of plate-bound anti-CD3 (OKT3, eBioscience, San Diego, CA) or an isotype control. Supernatants were harvested for cytokine quantification by enzyme-linked immunosorbent assay (ELISA). For proliferation assays, bromodeoxyuridine (BrDU) was added in the last 12 hrs according to the manufacturer's instruction. Informed written consent was obtained from all healthy donors involved in this study.

### ELISA

The ELISA kit for IL-8 was purchased from R&D Systems (Minneapolis, MN) and the cell proliferation BrDU ELISA kit from Roche (Mississauga, ON, Canada). ELISAs were done according to the manufacturer's instruction. The IFN-γ ELISA was completed as previously described [43].

### Proteome Profiler Analysis

The proteome profiler *Human Cytokine Array Panel A Array Kit* (R&D Systems) was used according to the manufacturer's instructions. Briefly, the membranes were incubated with conditioned media from LNCaP cells stimulated with R1881, as previously described, in the presence of the supplied antibody cocktail. Following washes and incubation with a Streptavidin-HRP buffer, positive signal was revealed using ECL reagent.

### Statistics

Statistical analysis was performed using SPSS software 11.0 (SPSS Inc., Chicago, Il). The non-parametric Mann-Whitney U test was used to show statistically significant differences.

## Supporting Information

Table S1 Correlations between ARG2 expression and clinico-pathological markers.
(0.05 MB XLS)

Table S2 Correlations between ARG2 expression and immune cell infiltration.
(0.05 MB XLS)

Figure S1 Androgen stimulation of PCa cells. A) LNCaP cells (left panels) and 22RV1 (right panels) were stimulated over a period of 72 hours with 10 nM R1881 following a 72 hour incubation period in charcoal-stripped media and the gene expression of Prostate-Specific Antigen (PSA), a positive control for R1881 stimulation, was analyzed by qPCR. Note that the ARG2 gene expression presented in Figure 1A correlated with the higher androgen sensibility of LNCaP cells compared to 22RV1 as exemplified by the mRNA expression of PSA. B) Expression of ARG1 and ARG2 determined by Western blot in LNCaP, Du145 and PC3 cells stimulated with R1881 for 72 hours as previously described. Note the absence of ARG1 and ARG2 in the two HR PCa cell lines, DU145 and PC3.
(0.72 MB TIF)

Figure S2 ARG1 and ARG2 induction following IL-8 stimulation. A) Positive signals from the Proteome Profiler™ were quantified by densitometry using Quantity One software (Bio-Rad). Ethanol control (gray bars) and R1881 (black bars). B) Arginase activity of LNCaP cells transfected with siCTRL, siAR or siIL-8 and then stimulated with R1881 was quantified in mU/mg of proteins. Same representative experiment as presented in Figure 2F, (n = 3). C) Increased ARG2 gene expression at 24 hours following IL-8 stimulation. LNCaP were stimulated with IL-8 as previously described. Ran served as the loading control. D) LNCaP cells were plated in charcoal-stripped serum supplemented phenol red-free RPMI media for 72 hours and subsequently for 24 hours in serum-free, phenol red-free RPMI. Cells were then stimulated for 72 hours with 10 nM R1881 or with 5 nM of IL-8 in serum-free, phenol red-free RPMI. ARG1 and ARG2 expression levels were detected by Western blot. Representative experiment is shown, (n = 2).
(1.43 MB TIF)

## References

[1] Jemal A, Siegel R, Ward E, Hao Y, Xu J (2008). Cancer statistics, 2008.. CA Cancer J Clin.

[2] Grossmann ME, Huang H, Tindall DJ (2001). Androgen receptor signaling in androgen-refractory prostate cancer.. J Natl Cancer Inst.

[3] Montironi R, Schulman CC (1998). Pathological changes in prostate lesions after androgen manipulation.. J Clin Pathol.

[4] Chang SS, Kibel AS (2009). The role of systemic cytotoxic therapy for prostate cancer.. BJU Int.

[5] Miller AM, Pisa P (2007). Tumor escape mechanisms in prostate cancer.. Cancer Immunol Immunother.

[6] Mumenthaler SM, Yu H, Tze S, Cederbaum SD, Pegg AE (2008). Expression of arginase II in prostate cancer.. Int J Oncol.

[7] Bronte V, Kasic T, Gri G, Gallana K, Borsellino G (2005). Boosting antitumor responses of T lymphocytes infiltrating human prostate cancers.. J Exp Med.

[8] Bronte V, Zanovello P (2005). Regulation of immune responses by L-arginine metabolism.. Nat Rev Immunol.

[9] Mercader M, Bodner BK, Moser MT, Kwon PS, Park ES (2001). T cell infiltration of the prostate induced by androgen withdrawal in patients with prostate cancer.. Proc Natl Acad Sci U S A.

[10] Gannon PO, Poisson AO, Delvoye N, Lapointe R, Mes-Masson AM (2009). Characterization of the intra-prostatic immune cell infiltration in androgen-deprived prostate cancer patients.. J Immunol Methods.

[11] Yamanaka H, Kirdani RY, Saroff J, Murphy GP, Sandberg AA (1975). Effects of testosterone and prolactin on rat prostatic weight, 5alpha-reductase, and arginase.. Am J Physiol.

[12] Manteuffel-Cymborowska M, Chmurzynska W, Peska M, Grzelakowska-Sztabert B (1995). Arginine and ornithine metabolizing enzymes in testosterone-induced hypertrophic mouse kidney.. Int J Biochem Cell Biol.

[13] Levillain O, Diaz JJ, Blanchard O, Dechaud H (2005). Testosterone down-regulates ornithine aminotransferase gene and up-regulates arginase II and ornithine decarboxylase genes for polyamines synthesis in the murine kidney.. Endocrinology.

[14] Lu S, Wang A, Dong Z (2007). A novel synthetic compound that interrupts androgen receptor signaling in human prostate cancer cells.. Mol Cancer Ther.

[15] Munder M (2009). Arginase: an emerging key player in the mammalian immune system.. Br J Pharmacol.

[16] Munder M, Eichmann K, Moran JM, Centeno F, Soler G (1999). Th1/Th2-regulated expression of arginase isoforms in murine macrophages and dendritic cells.. J Immunol.

[17] Seaton A, Scullin P, Maxwell PJ, Wilson C, Pettigrew J (2008). Interleukin-8 signaling promotes androgen-independent proliferation of prostate cancer cells via induction of androgen receptor expression and activation.. Carcinogenesis.

[18] Lin MF, Lee MS, Garcia-Arenas R, Lin FF (2000). Differential responsiveness of prostatic acid phosphatase and prostate-specific antigen mRNA to androgen in prostate cancer cells.. Cell Biol Int.

[19] Mumenthaler SM, Rozengurt N, Livesay JC, Sabaghian A, Cederbaum SD (2008). Disruption of arginase II alters prostate tumor formation in TRAMP mice.. Prostate.

[20] Kee K, Vujcic S, Merali S, Diegelman P, Kisiel N (2004). Metabolic and antiproliferative consequences of activated polyamine catabolism in LNCaP prostate carcinoma cells.. J Biol Chem.

[21] Xu Q, Baker BS, Tata JR (1993). Developmental and hormonal regulation of the Xenopus liver-type arginase gene.. Eur J Biochem.

[22] Olszewska A, Krol K, Weglenski P, Dzikowska A (2007). Arginine catabolism in Aspergillus nidulans is regulated by the rrmA gene coding for the RNA-binding protein.. Fungal Genet Biol.

[23] Harris BE, Pretlow TP, Bradley EL, Whitehurst GB, Pretlow TG, 2nd (1983). Arginase activity in prostatic tissue of patients with benign prostatic hyperplasia and prostatic carcinoma.. Cancer Res.

[24] Keskinege A, Elgun S, Yilmaz E (2001). Possible implications of arginase and diamine oxidase in prostatic carcinoma.. Cancer Detect Prev.

[25] Pretlow TG, 2nd, Harris BE, Bradley EL, Bueschen AJ, Lloyd KL (1985). Enzyme activities in prostatic carcinoma related to Gleason grades.. Cancer Res.

[26] Singh R, Pervin S, Karimi A, Cederbaum S, Chaudhuri G (2000). Arginase activity in human breast cancer cell lines: N(omega)-hydroxy-L-arginine selectively inhibits cell proliferation and induces apoptosis in MDA-MB-468 cells.. Cancer Res.

[27] Buga GM, Wei LH, Bauer PM, Fukuto JM, Ignarro LJ (1998). NG-hydroxy-L-arginine and nitric oxide inhibit Caco-2 tumor cell proliferation by distinct mechanisms.. Am J Physiol.

[28] Tate DJ, Vonderhaar DJ, Caldas YA, Metoyer T, Patterson JRt (2008). Effect of arginase II on L-arginine depletion and cell growth in murine cell lines of renal cell carcinoma.. J Hematol Oncol.

[29] Brat DJ, Bellail AC, Van Meir EG (2005). The role of interleukin-8 and its receptors in gliomagenesis and tumoral angiogenesis.. Neuro Oncol.

[30] Araki S, Omori Y, Lyn D, Singh RK, Meinbach DM (2007). Interleukin-8 is a molecular determinant of androgen independence and progression in prostate cancer.. Cancer Res.

[31] Murphy C, McGurk M, Pettigrew J, Santinelli A, Mazzucchelli R (2005). Nonapical and cytoplasmic expression of interleukin-8, CXCR1, and CXCR2 correlates with cell proliferation and microvessel density in prostate cancer.. Clin Cancer Res.

[32] Lessard L, Saad F, Le Page C, Diallo JS, Peant B (2007). NF-kappaB2 processing and p52 nuclear accumulation after androgenic stimulation of LNCaP prostate cancer cells.. Cell Signal.

[33] Diallo JS, Betton B, Parent N, Peant B, Lessard L (2008). Enhanced killing of androgen-independent prostate cancer cells using inositol hexakisphosphate in combination with proteasome inhibitors.. Br J Cancer.

[34] Pfaffl MW (2001). A new mathematical model for relative quantification in real-time RT-PCR.. Nucleic Acids Res.

[35] Le Page C, Koumakpayi IH, Lessard L, Saad F, Mes-Masson AM (2005). Independent role of phosphoinositol-3-kinase (PI3K) and casein kinase II (CK-2) in EGFR and Her-2-mediated constitutive NF-kappaB activation in prostate cancer cells.. Prostate.

[36] Grandvaux N, Gaboriau F, Harris J, tenOever BR, Lin R (2005). Regulation of arginase II by interferon regulatory factor 3 and the involvement of polyamines in the antiviral response.. FEBS J.

[37] Le Page C, Koumakpayi IH, Alam-Fahmy M, Mes-Masson AM, Saad F (2006). Expression and localisation of Akt-1, Akt-2 and Akt-3 correlate with clinical outcome of prostate cancer patients.. Br J Cancer.

[38] Diallo JS, Aldejmah A, Mouhim AF, Peant B, Fahmy MA (2007). NOXA and PUMA expression add to clinical markers in predicting biochemical recurrence of prostate cancer patients in a survival tree model.. Clin Cancer Res.

[39] Gannon PO, Koumakpayi IH, Le Page C, Karakiewicz PI, Mes-Masson AM (2008). Ebp1 expression in benign and malignant prostate.. Cancer Cell Int.

[40] Koumakpayi IH, Diallo JS, Le Page C, Lessard L, Gleave M (2006). Expression and nuclear localization of ErbB3 in prostate cancer.. Clin Cancer Res.

[41] Gannon PO, Alam Fahmy M, Begin LR, Djoukhadjian A, Filali-Mouhim A (2006). Presence of prostate cancer metastasis correlates with lower lymph node reactivity.. Prostate.

[42] Gopalakrishnan V, Burton PJ, Blaschke TF (1996). High-performance liquid chromatographic assay for the quantitation of L-arginine in human plasma.. Anal Chem.

[43] Godin-Ethier J, Pelletier S, Hanafi LA, Gannon PO, Forget MA (2009). Human activated T lymphocytes modulate IDO expression in tumors through Th1/Th2 balance.. J Immunol.

