# Supplementary figures and images for: Androgen-Regulated Expression of Arginase 1, Arginase 2 and Interleukin-8 in Human Prostate Cancer
Source: PLoS One. 2010 Aug 11;5(8):e12107. doi: 10.1371/journal.pone.0012107 (PMC2920336; doi:10.1371/journal.pone.0012107)

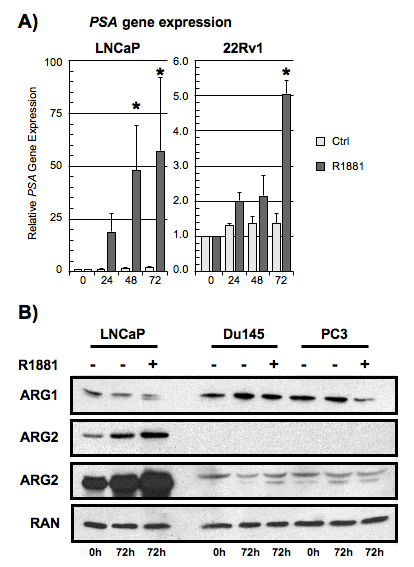

Supplement: Figure S1 — Androgen stimulation of PCa cells. A) LNCaP cells (left panels) and 22RV1 (right panels) were stimulated over a period of 72 hours with 10 nM R1881 following a 72 hour incubation period in charcoal-stripped media and the gene expression of Prostate-Specific Antigen (PSA), a positive control for R1881 stimulation, was analyzed by qPCR. Note that the ARG2 gene expression presented in Figure 1A correlated with the higher androgen sensibility of LNCaP cells compared to 22RV1 as exemplified by the mRNA expression of PSA. B) Expression of ARG1 and ARG2 determined by Western blot in LNCaP, Du145 and PC3 cells stimulated with R1881 for 72 hours as previously described. Note the absence of ARG1 and ARG2 in the two HR PCa cell lines, DU145 and PC3. (0.72 MB TIF) [file pone.0012107.s003.tif]

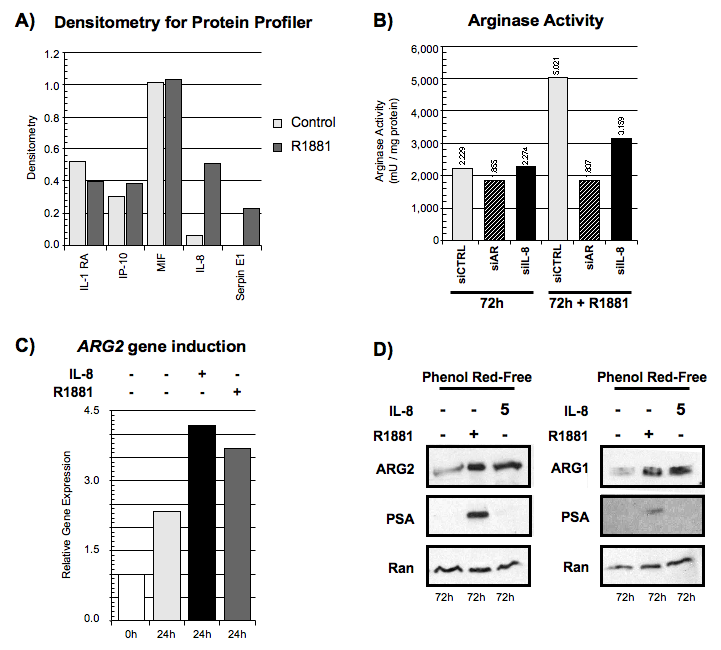

Supplement: Figure S2 — ARG1 and ARG2 induction following IL-8 stimulation. A) Positive signals from the Proteome Profiler™ were quantified by densitometry using Quantity One software (Bio-Rad). Ethanol control (gray bars) and R1881 (black bars). B) Arginase activity of LNCaP cells transfected with siCTRL, siAR or siIL-8 and then stimulated with R1881 was quantified in mU/mg of proteins. Same representative experiment as presented in Figure 2F, (n = 3). C) Increased ARG2 gene expression at 24 hours following IL-8 stimulation. LNCaP were stimulated with IL-8 as previously described. Ran served as the loading control. D) LNCaP cells were plated in charcoal-stripped serum supplemented phenol red-free RPMI media for 72 hours and subsequently for 24 hours in serum-free, phenol red-free RPMI. Cells were then stimulated for 72 hours with 10 nM R1881 or with 5 nM of IL-8 in serum-free, phenol red-free RPMI. ARG1 and ARG2 expression levels were detected by Western blot. Representative experiment is shown, (n = 2). (1.43 MB TIF) [file pone.0012107.s004.tif]
